# Agreement Between Arterial Carbon Dioxide Levels With End-Tidal Carbon Dioxide Levels and Associated Factors in Children Hospitalized With Traumatic Brain Injury

**DOI:** 10.1001/jamanetworkopen.2019.9448

**Published:** 2019-08-16

**Authors:** Jen-Ting Yang, Scott L. Erickson, Elizabeth Y. Killien, Brianna Mills, Abhijit V. Lele, Monica S. Vavilala

**Affiliations:** 1 Harborview Injury Prevention and Research Center, University of Washington, Seattle; 2 Department of Anesthesiology & Pain Medicine, University of Washington, Seattle; 3 Department of Epidemiology, University of Washington, Seattle; 4 Department of Pediatrics, University of Washington, Seattle

## Abstract

**Importance:**

Alterations in the partial pressure of carbon dioxide, arterial (Paco_2_) can affect cerebral perfusion after traumatic brain injury. End-tidal carbon dioxide (EtCO_2_) monitoring is a noninvasive tool used to estimate Paco_2_ values.

**Objective:**

To examine the agreement between Paco_2_ and EtCO_2_ and associated factors in children with traumatic brain injury.

**Design, Setting, and Participants:**

A secondary analysis was conducted using data from a prospective cohort study of 137 patients younger than 18 years with traumatic brain injury who were admitted to the pediatric intensive care unit of a level I trauma center between May 1, 2011, and July 31, 2017. Analysis was performed from December 17, 2018, to January 10, 2019.

**Main Outcomes and Measures:**

The closest EtCO_2_ value obtained within 30 minutes of a Paco_2_ value and the closest systolic blood pressure value obtained within 60 minutes prior to a Paco_2_ value during the first 24 hours after admission were recorded. The main outcome of Paco_2_-EtCO_2_ agreement was defined as Paco_2_ between 0 and 5 mm Hg greater than the paired EtCO_2_ value, and it was determined using Bland-Altman analysis, Passing and Bablok regression, and the Pearson correlation coefficient. Multivariable regression models determined which factors were associated with agreement.

**Results:**

The analysis included 137 patients (34 girls and 103 boys; mean [SD] age, 10.0 [6.3] years) and 445 paired Paco_2_-EtCO_2_ data points. On average, Paco_2_ was 2.7 mm Hg (95% limits of agreement, –11.3 to 16.7) higher than EtCO_2_. Overall, 187 of all Paco_2_-EtCO_2_ pairs (42.0%) agreed. There was larger variation in the Paco_2_-EtCO_2_ difference during the first 8 hours compared with 9 to 24 hours after admission to the pediatric intensive care unit. Development of pediatric acute respiratory distress syndrome within 24 hours of admission was associated with a lower likelihood of Paco_2_-EtCO_2_ agreement (adjusted odds ratio, 0.20; 95% CI, 0.08-0.51) compared with no development of pediatric acute respiratory distress syndrome. A diagnosis of pediatric acute respiratory distress syndrome 1 to 7 days after admission was associated with a larger first-day Paco_2_-EtCO_2_ difference compared with those who never developed pediatric acute respiratory distress syndrome (mean [SD] difference, 4.48 [3.70] vs 0.46 [5.50] mm Hg).

**Conclusions and Relevance:**

In this study of pediatric traumatic brain injury, Paco_2_-EtCO_2_ agreement was low, especially among patients with pediatric acute respiratory distress syndrome. Low Paco_2_-EtCO_2_ agreement early in hospitalization may be associated with future development of pediatric acute respiratory distress syndrome. Data on EtCO_2_ should not be substituted for data on Paco_2_ during the first 24 hours.

## Key Points

**Question:**

Is end-tidal carbon dioxide (EtCO_2_) a reliable surrogate for partial pressure of carbon dioxide, arterial (Paco_2_) in children admitted to the intensive care unit with traumatic brain injury?

**Findings:**

In this secondary analysis of a prospective cohort study that included 445 paired Paco_2_-EtCO_2_ values from 137 patients, only 42.0% of Paco_2_ values were within 0 to 5 mm Hg of paired EtCO_2_ values. Development of pediatric acute respiratory distress syndrome within 24 hours after admission was associated with significantly lower likelihood of Paco_2_-EtCO_2_ agreement than was no development of pediatric acute respiratory distress syndrome.

**Meaning:**

This study suggests that EtCO_2_ values are not a reliable substitute for Paco_2_ values during the first 24 hours after pediatric traumatic brain injury, especially in the presence of pediatric acute respiratory distress syndrome.

## Teaser

This secondary analysis of a cohort study examines agreement between partial pressure of carbon dioxide, arterial (Paco_2_) and end-tidal carbon dioxide (EtCO_2_) and associated factors in children with traumatic brain injury (TBI).

## Introduction

Globally, traumatic brain injury (TBI) is a leading cause of pediatric morbidity and mortality.^1,2^ During TBI care, arterial sampling allows for the adjustment of the partial pressure of carbon dioxide, arterial (Paco_2_) to help regulate cerebral perfusion. However, arterial sampling can be challenging in children, and complications are not uncommon.^3,4^ Capnography allows for noninvasive, continuous measurement of end-tidal carbon dioxide (EtCO_2_) with lower cost and fewer complications compared with arterial cannulation, and it is widely used in clinical pediatric practice as a substitute for arterial cannulation.^5^ End-tidal CO_2_ reasonably estimates Paco_2_ in healthy adults.^4,5,6^ A mean difference of 0 to 5 mm Hg between Paco_2_ and EtCO_2_ is generally accepted as good agreement, and good agreement has been demonstrated in patients during general anesthesia who are undergoing prolonged neurosurgical procedures, in clinically stable patients in the pediatric intensive care unit (PICU), and in adult patients with TBI without hypotension, metabolic acidosis, or severe lung injury.^7,8,9,10^ However, the utility of EtCO_2_ as a substitute for Paco_2_ in critically injured children with TBI has not been examined, to our knowledge.

Recently, the Brain Trauma Foundation published the third edition of medical management guidelines for severe pediatric TBI, recommending avoidance of prophylactic hyperventilation and Paco_2_ less than 30 mm Hg in the initial 48 hours after admission.^11^ A recent study showed that adhering to this recommendation improved discharge outcomes.^12^ The Brain Trauma Foundation does not, however, include a recommendation either for or against the use of EtCO_2_ measurements because of the lack of data comparing Paco_2_ with EtCO_2_ to prevent unwanted hypocarbia and hypercarbia. To evaluate the validity of using EtCO_2_ as an indicator of Paco_2_ in children and adolescents hospitalized with TBI, we performed a secondary analysis of a prospective cohort study to describe the agreement between Paco_2_ and EtCO_2_ early after TBI and determine the factors associated with Paco_2_-EtCO_2_ agreement.

## Methods

After approval by the University of Washington Human Subjects Division, from December 17, 2018, to January 10, 2019, we conducted a secondary analysis of data from the recently published Pediatric Guideline Adherence and Outcomes program study.^12^ Waiver of consent was granted by the University of Washington Institutional Review Board for the parent project because this research was a secondary data analysis that was deemed to involve no more than minimal risk to participants and waiver would not adversely affect the rights and welfare of the participants. This study followed the Strengthening the Reporting of Observational Studies in Epidemiology (STROBE) reporting guideline.

### Study Participants and Setting

Harborview Medical Center is a 450-bed mixed level I adult and pediatric trauma hospital that serves the 5-state (Washington, Wyoming, Alaska, Montana, and Idaho) Pacific Northwest region. The 18-bed PICU admits approximately 120 pediatric trauma patients with TBI annually.

Study participants were patients younger than 18 years admitted to the PICU at Harborview Medical Center with TBI between May 1, 2011, and July 31, 2017. Patients were eligible if they (1) had 1 or more EtCO_2_ values recorded within 30 minutes of a Paco_2_ measurement during the first 24 hours after admission and (2) had 1 or more Paco_2_-EtCO_2_ pairs with a systolic blood pressure (SBP) measured at any point within the 60 minutes prior to the Paco_2_ value (eFigure 1 in the Supplement).

### Data Collection

Data on demographic characteristics, mechanisms of injury, and injury severity measures were obtained from the Harborview Trauma Registry. Ascertainment of diagnosis of TBI via computed tomography, surgical procedures performed, Paco_2_, EtCO_2_, SBP, oxygenation index, oxygenation saturation index, and Glasgow Coma Scale score were abstracted from the electronic health record. Race and ethnicity were self-reported.

### Measurement of EtCO_2_ and Paco_2_ Levels

Clinical data collected during the first 24 hours of clinical care after admission to the PICU were examined. Paco_2_ measured via arterial blood gas was entered into the electronic health record by laboratory personnel. End-tidal CO_2_ was measured via Medtronic capnography and manually entered by PICU nurses or respiratory therapists into the electronic health record at a minimum of every hour. Data on SBP associated with EtCO_2_ and Paco_2_ levels were recorded with a digital sphygmomanometer or by invasive arterial monitoring and were automatically entered from SpaceLabs monitoring system at least every hour, or more frequently as dictated by clinical care. All paired data points were collected while the patients were intubated and mechanically ventilated.

### Ascertainment of Pediatric Acute Respiratory Distress Syndrome 

Patients were determined to have pediatric acute respiratory distress syndrome (PARDS) if they met Pediatric Acute Lung Injury Consensus Conference PARDS criteria within 7 days of their injury.^13^ Patients with at least 3 qualifying values of the oxygenation index or the oxygenation saturation index during a minimum of 6 hours were determined to meet oxygenation criteria for PARDS. Medical record abstraction was performed for all patients meeting oxygenation criteria to evaluate for the presence of a new parenchymal infiltrate detected on a chest radiograph (based on review by the attending radiologist and secondary review by a pediatric intensivist [E.Y.K.]) and for the origin of edema (based on documentation by the attending physician).

### Alignment of EtCO_2_ and Paco_2_ Data With SBP Data

For every arterial blood gas measurement obtained during routine medical care, the nearest EtCO_2_ value recorded within 30 minutes was used to create 1 Paco_2_-EtCO_2_ data pair. The nearest SBP value within 60 minutes prior to the Paco_2_ value being measured was then added to create a Paco_2_-EtCO_2_-SBP trio. Values measured during apnea trials for brain death were excluded because the patients were no longer receiving routine TBI care.

### Definition of Paco_2_-EtCO_2_ Agreement

End-tidal CO_2_ measured by capnography is a mixture of relatively CO_2_-rich alveolar gas diffused from capillaries with CO_2_-poor gas in ventilated lung regions with poor perfusion (ie, dead space). Dead space includes the components of the respiratory system that are ventilated but do not participate in gas exchange and alveoli that receive ventilation but are not perfused because of physiologic or pathologic factors, as well as the airways and breathing apparatus. The Paco_2_ value is typically 2 to 5 mm Hg higher than the EtCO_2_ value under physiologically stable conditions.^14^ Therefore, Paco_2_-EtCO_2_ pairs were categorized as either in agreement, if the Paco_2_ value was between 0 and 5 mm Hg greater than its paired EtCO_2_ value, or not in agreement.

### Choice and Coding of Explanatory Variables

Potential explanatory variables were determined a priori based on literature review and research team discussion, and they included severity of chest injury, severity of nonhead-nonchest injury, presence of PARDS (within 24 hours or 1-7 days after admission), hypotension, patient age, time since admission, and the time difference between Paco_2_ measurement and when EtCO_2_ was recorded.

The presence of severe chest injury or nonhead-nonchest injury with a region-specific Abbreviated Injury Score greater than 2 was coded as a binary variable. Hypotension was defined as SBP less than 70 mm Hg +2 × (patient age in years) coded as a binary variable. The PARDS status for each patient was categorized as follows: did not develop within the first week of PICU admission, developed within 24 hours of PICU admission, or developed after 24 hours but within 1 week of PICU admission. Time after admission was dichotomized into 0 to 8 hours and 9 to 24 hours, and age was categorized into younger than 1, 1 to 4, 5 to 9, 10 to 14, and 15 to 17 years.

### Statistical Analysis

We used descriptive statistics to examine clinical injury characteristics. Agreement between Paco_2_ and EtCO_2_ was assessed for the overall study population and stratified by PARDS status at 24 hours using Bland-Altman plots with limits of agreement adjusted for repeated measures.^15^ We also assessed the proportion of Paco_2_-EtCO_2_ pairs with Paco_2_ values 0 to 5 mm Hg greater than their paired EtCO_2_ value.

The characteristics that may have affected the likelihood of agreement between measures were initially assessed descriptively, comparing Paco_2_-EtCO_2_ pairs in agreement with pairs that did not agree. Stratified Bland-Altman plots were created to visually compare trends in agreement across levels of age and whether patients developed PARDS within the first 24 hours and within 1 to 7 days of PICU admission. We assessed constant and proportional differences between Paco_2_ and EtCO_2_ within each Paco_2_-EtCO_2_ pair, and we estimated Pearson correlation coefficients using Passing and Bablok regression and Deming regression.^16,17^

To determine the characteristics associated with agreement between Paco_2_ and EtCO_2_, we used univariate logistic regression models clustered by patient; to minimize confounding, we used multivariable logistic regression models clustered by patient adjusted for age, PARDS within the first 24 hours of PICU admission, severe TBI, severe chest injury, severe nonhead-nonchest injury, and shock. Anatomic dead space in the pediatric population could have considerable variation and may affect Paco_2_-EtCO_2_ agreement. Although we were not able to measure dead space individually, we chose to adjust for age and cluster by patient to address this issue. To account for the nonindependence of data points within patients, we used a fixed-effects model to assess the association between hypotension (the only time-varying a priori selected explanatory variable) and Paco_2_-EtCO_2_ agreement within individual patients.

We described the temporal trends of Paco_2_ and EtCO_2_ difference graphically to assess the association between time after PICU admission and Paco_2_-EtCO_2_ agreement. To explore how the median first-day Paco_2_-EtCO_2_ difference after PICU admission differed by the timing of PARDS diagnosis, we also constructed side-by-side boxplots of patients who had no PARDS, received a diagnosis within 24 hours of PICU admission, or received a diagnosis 1 to 7 days after PICU admission. Two-sample *t* tests were used to determine if there were statistically significant differences in the mean Paco_2_-EtCO_2_ difference between these groups. The 2 analyses were repeated among patients who survived the first 24 hours of PICU admission to examine the association of early death with our findings.

To explore the association of Paco_2_-EtCO_2_ differences with subsequent PARDS among patients who had not developed PARDS within the first 24 hours, we performed a receiver operating characteristic curve analysis and examined the sensitivity and specificity of various cutoff values. All statistical analyses were performed using R, version 3.5.1 (R Project for Statistical Computing), and Stata, version 14.2 package (StataCorp),^18,19^ and analyses were approved by the study epidemiologist (B.M.).

## Results

Of the 199 potentially eligible patients, 137 (103 boys and 34 girls) met the inclusion criteria; 62 patients and 6 Paco_2_-EtCO_2_ pairs were excluded owing to a lack of data within the predefined measurement time window (eFigure 1 in the Supplement) or the results of apnea testing for brain death. The final analytic cohort contributed 445 paired Paco_2_-EtCO_2_ data points (Table 1). The median number of Paco_2_-EtCO_2_ pairs per patient was 3 (interquartile range, 2-4) (eFigure 2 in the Supplement). Compared with patients who were excluded, patients who met the inclusion criteria had higher injury severity scores and more commonly underwent craniotomy (eTable 1 in the Supplement). Paco_2_ agreed with its paired EtCO_2_ value in 187 Paco_2_-EtCO_2_ pairs (42.0%).

**Table 1.** Cohort Characteristics of Hospitalized Children With TBI by Agreement Status Between Paco_2_ and EtCO_2_ During the First 24 Hours After PICU Admission^a^

Characteristic	Total Cohort (N = 137)	Paco_2_-EtCO_2_		
		Pairs (N = 445)	Pairs in Agreement (n = 187)	Pairs Not in Agreement (n = 258)
Points per patient, median (IQR)	3 (2-4)	3 (2-4)	2 (1-3)	2 (1-3)
Age, y				
<1	8 (5.8)	23 (5.2)	9 (4.8)	14 (5.4)
1-4	32 (23.4)	113 (25.4)	44 (23.5)	69 (26.7)
5-9	21 (15.3)	56 (12.6)	19 (10.2)	37 (14.3)
10-14	15 (10.9)	60 (13.5)	29 (15.5)	31 (12.0)
15-18	61 (44.5)	193 (43.4)	86 (46.0)	107 (41.5)
TBI severity at PICU admission				
Mild, GCS score >13	2 (1.5)	9 (2.0)	4 (2.1)	5 (1.9)
Moderate, GCS score 9-13	12 (8.8)	34 (7.6)	10 (5.4)	24 (9.3)
Severe, GCS <9	123 (89.8)	402 (90.4)	173 (92.5)	229 (88.8)
AIS score, median (IQR)				
Head	5.00 (4.00-5.00)	5.00 (4.00-5.00)	5.00 (4.00-5.00)	5.00 (4.00-5.00)
Chest	0.00 (0.00-3.00)	0.00 (0.00-3.00)	0.00 (0.00-3.00)	0.00 (0.00-3.00)
Nonhead highest AIS score, median (IQR)	2.00 (1.00-3.00)	2.00 (1.00-3.00)	2.00 (1.00-3.00)	2.00 (1.00-4.00)
Injury severity score, median (IQR)	30.00 (25.00-38.00)	30.00 (26.00-41.00)	30.00 (26.00-41.00)	30.00 (25.00-41.75)
PARDS				
First 24 h of admission	14 (10.2)	67 (15.1)	11 (5.9)	56 (21.7)
1-7 d after admission	22 (16.1)	92 (20.7)	45 (24.1)	47 (18.2)
No PARDS within 7 d of admission	101 (73.7)	286 (64.3)	131 (70.0)	155 (60.1)
EtCO_2_ within 10 min of Paco_2_	NA	186 (41.8)	77 (41.2)	109 (42.2)
Paco_2_ within 30 min of hypotension	NA	64 (14.4)	26 (13.9)	38 (14.7)
Survived 24 h of PICU admission	127 (92.7)	420 (94.4)	177 (94.7)	243 (94.2)

Abbreviations: AIS, Abbreviated Injury Scale; EtCO_2_, end-tidal carbon dioxide; GCS, Glasgow Coma Scale; IQR, interquartile range; NA, not applicable; Paco_2_, partial pressure of carbon dioxide, arterial; PARDS; pediatric acute respiratory distress syndrome; PICU, pediatric intensive care unit; TBI, traumatic brain injury.

^a^ Data are presented as number (percentage) of patients unless otherwise indicated.

A total of 67 of the 445 Paco_2_-EtCO_2_ pairs (15.1%) were recorded for patients who experienced PARDS within 24 hours of PICU admission (Table 1), and 92 of the 445 Paco_2_-EtCO_2_ pairs (20.7%) were recorded for patients who developed PARDS between 1 and 7 days after PICU admission. Compared with data pairs in agreement, pairs not in agreement were more commonly from patients with PARDS diagnosed within 24 hours of admission (56 of 258 [21.7%] vs 11 of 187 [5.9%]).

**Figure 1.**
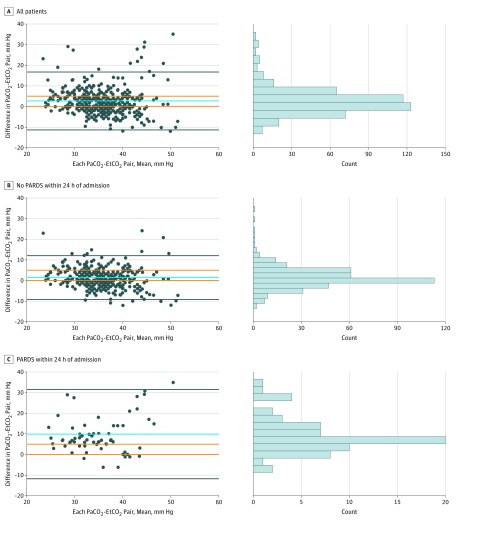
Bland-Altman Analysis Comparing Partial Pressure of Carbon Dioxide, Arterial (Paco_2_) and End-Tidal Carbon Dioxide (EtCO_2_) A, Bland-Altman analysis comparing Paco_2_ and EtCO_2_ for all patients (445 Paco_2_-EtCO_2_ pairs for 137 patients; 187 [42.0%] of these pairs had EtCO_2_ values within 5 mm Hg of paired Paco_2_). B, Bland-Altman analysis comparing Paco_2_ and EtCO_2_ for those who did not develop pediatric acute respiratory distress syndrome (PARDS) within 24 hours of pediatric intensive care unit (PICU) admission (376 Paco_2_-EtCO_2_ pairs for 123 patients; 176 [46.8%] of these pairs had EtCO_2_ values within 5 mm Hg of paired Paco_2_). C, Bland-Altman analysis comparing Paco_2_ and EtCO_2_ for those who developed PARDS within 24 hours of PICU admission (67 Paco_2_-EtCO_2_ pairs for 14 patients; 11 [16.4%] of these pairs had EtCO_2_ values within 5 mm Hg of paired Paco_2_). Bias is represented by the blue line. The limits of agreement are represented by the black lines and adjust for repeated measures. A priori limits of acceptable agreement are represented by orange lines. Marginal histograms describe distribution of values between pairs.

Among all 445 Paco_2_-EtCO_2_ pairs, Paco_2_ was on average 2.7 mm Hg (95% limits of agreement, –11.3 to 16.7 mm Hg) higher than its paired EtCO_2_ value (Figure 1A). Paco_2_ was 0 to 5 mm Hg greater than its paired EtCO_2_ value in 187 of 445 Paco_2_-EtCO_2_ pairs (42.0%). A total of 43 patients (31.4%) had at least 1 paired data point with disagreement. Among the 378 Paco_2_-EtCO_2_ pairs obtained from patients who did not experience PARDS within the first 24 hours of admission, Paco_2_ was on average 1.4 mm Hg (95% limits of agreement, –9.2 to 12.0 mm Hg) higher than EtCO_2_ (Figure 1B). Paco_2_ was 0 to 5 mm Hg greater than its paired EtCO_2_ value in 176 of 378 pairs (46.6%). Among the 67 Paco_2_-EtCO_2_ pairs obtained from patients who developed PARDS within first 24 hours of admission, Paco_2_ was on average 9.9 mm Hg (95% limits of agreement, –11.1 to 30.8 mm Hg) higher than its paired EtCO_2_ value (Figure 1C). Paco_2_ was 0 to 5 mm Hg greater than its paired EtCO_2_ value in 11 of 67 pairs (16.4%).

**Figure 2.**
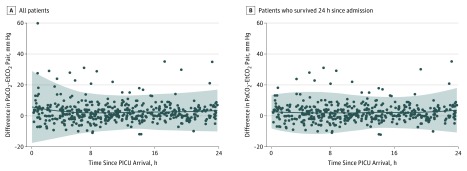
Paired Differences Between Partial Pressure of Carbon Dioxide, Arterial (Paco_2_) and End-Tidal Carbon Dioxide (EtCO_2_) by Hours Since Pediatric Intensive Care Unit (PICU) Admission A, Circles represent 445 Paco_2_-EtCO_2_ pairs for all 137 patients. B, Circles represent 420 Paco_2_-EtCO_2_ pairs for the 127 patients who survived 24 hours since PICU admission. Solid line indicates mean difference between Paco_2_ and EtCO_2_ by hour since PICU admission. Sharded areas indicate 95% prediction interval of the mean difference between Paco_2_ and EtCO_2_.

The range of measures in which Paco_2_ and EtCO_2_ are most similar are shown by Passing and Bablok regression (eFigure 3 in the Supplement), as well as by Deming regression (eFigure 4 in the Supplement). The Pearson correlation coefficient was 0.45. There was larger variation in the Paco_2_-EtCO_2_ difference during the first 8 hours since PICU admission compared with 9 to 24 hours after admission (Figure 2A); however, this pattern was not observed when examining only those patients who survived 24 hours from PICU admission (Figure 2B). Agreement by age groups is presented in eFigure 5 in the Supplement, where the mean Paco_2_-EtCO_2_ difference ranged from 1.29 to 5.78 mm Hg and Paco_2_-EtCO_2_ agreement ranged from 34% to 48%. Side-by-side boxplots of Paco_2_-EtCO_2_ differences within patients are plotted in eFigure 6 in the Supplement.

Table 2 and eTable 2 in the Supplement present the association between explanatory variables and Paco_2_-EtCO_2_ agreement estimated by unadjusted, adjusted multivariable, and fixed-effects models.^20^ Among the factors examined, first-day PARDS development was associated with a lower likelihood of Paco_2_-EtCO_2_ agreement when compared with no first-day PARDS (adjusted OR, 0.20; 95% CI, 0.08-0.51); there was no meaningful change in association after multivariable model adjustment.

**Table 2.** Factors Associated With Agreement Between Paco_2_ and EtCO_2_ and A Priori Selected Covariates Among 138 Pediatric Patients With Traumatic Brain Injury Admitted to the PICU

Variable	No. of Pairs in Agreement/Total No. of Pairs^a^	Odds Ratio (95% CI)		
		Unadjusted^b^	Adjusted^b^^,^^c^	Fixed-Effects Model^c^^,^^d^
PARDS within 24 h of PICU admission				
PARDS	11/67	0.23 (0.10-0.49)	0.20 (0.08-0.51)	NA
No PARDS	176/378			NA
PARDS 1-7 d after PICU admission				
PARDS	45/92	1.06 (0.50-2.22)	1.22 (0.61-2.45)	NA
No PARDS	131/286			NA
Timing of Paco_2_**-**EtCO_2_ pair relative to PICU admission				
Within first 8 h	64/169	0.76 (0.49-1.18)	NA	NA
9-24 h	123/276		NA	NA
Timing of Paco_2_-EtCO_2_ pairs relative to PICU admission among patients surviving 24 h				
Within first 8 h	60/155	0.80 (0.51-1.26)	NA	NA
9-24 h	117/265		NA	NA
Injury severity				
Head AIS score >2	180/415	1.34 (0.77-2.32)	1.49 (0.42-5.37)	NA
Head AIS score ≤2	7/30			NA
Chest AIS score >2	67/143	1.34 (0.77-2.32)	1.43 (0.81-2.54)	NA
Chest AIS score ≤2	120/304			NA
Maximum nonhead-nonchest AIS score >2	78/186	0.99 (0.60-1.64)	1.14 (0.66-1.98)	NA
Maximum nonhead-nonchest AIS score ≤2	109/259			NA
Hypotension status of Paco_2_**-**EtCO_2_ Pair^e^				
SBP <70 mm Hg + 2 × age	26/64	0.93 (0.53-1.66)	1.21 (0.65-2.24)	0.68 (0.30-1.51)
SBP ≥70 mm Hg + 2 × age	161/381			

Abbreviations: AIS, Abbreviated Injury Scale; EtCO_2_, end-tidal carbon dioxide; NA, not applicable; Paco_2_, partial pressure of carbon dioxide, arterial; PARDS, pediatric acute respiratory distress syndrome; PICU, pediatric intensive care unit; SBP, systolic blood pressure.

^a^ Agreement between Paco_2_ and EtCO_2_ defined as EtCO_2_ between 0 and 5 mm Hg, and 2 values were recorded within the same 30-minute interval.

^b^ Logistic regression clustered on patient was used in unadjusted and multivariable adjusted models.

^c^ Covariates were selected a priori for adjusted model, which included age, PARDS within 24 hours, chest injury, head injury, nonhead-nonchest injury, and shock.

^d^ Fixed-effect model accounted for presence of shock longitudinally within the same patient over time.

^e^ Per the *Advanced Trauma Life Support Manual*.^20^

**Figure 3.**
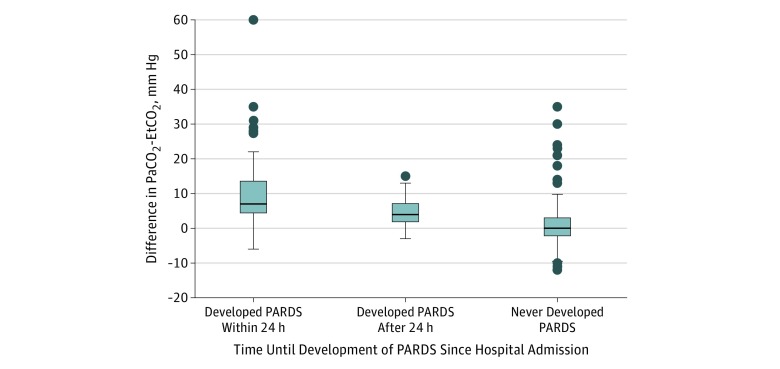
Boxplots of All Partial Pressure of Carbon Dioxide, Arterial (Paco_2_)–End-Tidal Carbon Dioxide (EtCO_2_) Differences by Pediatric Acute Respiratory Distress Syndrome (PARDS) Diagnosis Timing Data represent 445 Paco_2_-EtCO_2_ pairs for 137 patients. The median number of Paco_2_-EtCO_2_ pairs per patient was 3 (interquartile range, 2-4). Boxplots correspond to the timing of PARDS diagnosis: within 24 hours of pediatric intensive care unit (PICU) admission (67 pairs; n = 14), 1 to 7 days after PICU admission (92 pairs; n = 22), or never developed PARDS within 1 week of admission (286 pairs; n = 101). Based on 2-sample *t* tests assuming unequal variance, the mean Paco_2_-EtCO_2_ difference was 9.41 mm Hg greater (95% CI, 6.73-12.09 mm Hg; *P* < .001) among those who developed PARDS within 24 hours than those who never developed PARDS, was 4.02 mm Hg greater (95% CI, 3.02-5.01 mm Hg; *P* < .001) among those who developed PARDS after 24 hours than those who never developed PARDS, and was 5.39 mm Hg greater (95% CI, 2.68-8.11; *P* < .001) among those who developed PARDS within 24 hours than those who developed PARDS after 24 hours. Top and bottom whiskers represent ±2 times the interquartile range for each plot. Top and bottom borders of the boxes represent the 75th and 25th percentiles, respectively. Center horizontal line represents the median value. Circles represent outlier values as defined by points beyond 2 times the interquartile range.

The mean Paco_2_-EtCO_2_ difference during the first 24 hours of admission among those who developed PARDS within 24 hours was 9.41 mm Hg greater than those who never developed PARDS (95% CI, 6.73-12.10 mm Hg). The mean Paco_2_-EtCO_2_ difference during the first 24 hours of admission among patients who developed PARDS 1 to 7 days after admission was 4.02 mm Hg greater than those who never developed PARDS (95% CI, 3.02-5.01 mm Hg) (Figure 3). The mean Paco_2_-EtCO_2_ difference among those who developed PARDS within 24 hours was 5.39 mm Hg greater than those who developed PARDS 1 to 7 days after admission (95% CI, 2.68-8.11 mm Hg); restricting the sample to 24-hour survivors resulted in no meaningful change in findings. In the receiver operating characteristic curve analysis, the Paco_2_-EtCO_2_ difference among those who did not develop PARDS within 24 hours was associated with subsequent PARDS development between 1 and 7 days after admission (area under the curve, 0.78). In addition, a Paco_2_-EtCO_2_ difference of 3 mm Hg or greater had a sensitivity and specificity of 0.72 for the onset of PARDS between 1 and 7 days after PICU admission, given that PARDS had not developed within the first 24 hours (eTable 3 in the Supplement shows the sensitivity and specificity with different Paco_2_-EtCO_2_ difference cutoff values).

## Discussion

The main findings of this study of hospitalized pediatric patients with TBI are that (1) overall, fewer than half of all Paco_2_-EtCO_2_ pairs were in agreement, and correlation was only moderate; (2) the presence of PARDS in the first 24 hours of PICU admission was associated with a lower likelihood of Paco_2_-EtCO_2_ agreement; and (3) even if PARDS was not diagnosed in the first 24 hours, the median Paco_2_-EtCO2 differences were higher among a subset of patients who developed PARDS later in the first week after injury than among those who never developed PARDS. Together, these findings suggest that, despite the appeal of EtCO_2_ monitoring, EtCO_2_ data should not be substituted for Paco_2_ data early after pediatric TBI.

We used 3 complementary statistical approaches to understand the accuracy of EtCO_2_ as a Paco_2_ surrogate. First, we used Bland-Altman analysis, which revealed less than 50% agreement of EtCO_2_ with Paco_2_. Second, we used Passing and Bablok regression, which demonstrated that Paco_2_ and EtCO_2_ had positive constant and proportional differences between measurements. Finally, we used the Pearson correlation coefficient and demonstrated that Paco_2_ and EtCO_2_ were only moderately correlated. Collectively, these indicators demonstrated a moderate level of agreement and correlation between Paco_2_ and EtCO_2_. To our knowledge, this study is the first to use multiple measures of accuracy to address Paco_2_-EtCO_2_ agreement, and the results suggest a need to further define the role of capnography and the need to incorporate Paco_2_ measurements early after pediatric TBI.

Lee et al^10^ reported that among adults with TBI, 77.3% of Paco_2_ and EtCO_2_ values were within 5 mm Hg of each other. In contrast, our pediatric study demonstrated lower agreement and did not identify hypotension or severe chest injury as contributing factors to Paco_2_-EtCO_2_ agreement. Differences in the definition of Paco_2_-EtCO_2_ agreement and in the study design (PICU vs emergency department and timing of arterial blood measurement) may contribute to these differences in results. However, to reflect clinical practice, we defined agreement using a narrower range (Paco_2_-EtCO_2_ <5 mm Hg) and paired the data within clinically reasonable intervals, which likely decreased measured levels of agreement. Although the number of infants and young children in our study is too small to make definitive conclusions, the relatively larger amount of dead space in infants and children might further increase the Paco_2_-EtCO_2_ difference and lower Paco_2_-EtCO_2_ agreement.

Anatomic and physiologic dead space results in Paco_2_ values that are higher than EtCO_2_ values obtained simultaneously.^14^ Although there is generally no physiologic basis for why Paco_2_ would be lower than EtCO_2_, a negative Paco_2_-EtCO_2_ difference was observed among many of our Paco_2_-EtCO_2_ pairs. It is possible that this negative difference resulted from the accumulation of exhaled CO_2_ in ventilation circuitry prior to EtCO_2_ measurement or poor equipment calibration, but most likely it was due to our method of aligning Paco_2_-EtCO_2_ pairs during a period of up to 30 minutes, thus allowing the potential for intervening changes in patients’ clinical conditions to result in recorded peaks in EtCO_2_ values being paired with recorded dips in Paco_2_ values.

We specifically examined PARDS because children hospitalized with TBI are at risk for PARDS owing to a variety of factors, including intracranial hypertension, severe systemic inflammation, direct traumatic lung injury, and secondary lung injury from aspiration or pneumonia.^21,22^ It is thus reasonable to expect that many critically injured children with TBI, especially those with PARDS or other pulmonary pathologic characteristics, may have an elevated alveolar dead space fraction and thus lower Paco_2_-EtCO_2_ agreement. This study identified PARDS as an independent factor associated with a lower likelihood of Paco_2_-EtCO_2_ agreement, which provides new information on the utility of capnography among children with TBI with concurrent lung disease. These data also demonstrated that, for patients who had not received a diagnosis of PARDS during the first 24 hours of PICU admission, Paco_2_-EtCO_2_ differences were still greater among those who developed PARDS after the first 24 hours of admission compared with those who never developed PARDS, suggesting that patients may have had a ventilation-perfusion mismatch and worsening dead space fraction before meeting the full criteria for PARDS. Future work is needed to further evaluate Paco_2_-EtCO_2_ agreement as a potential early indicator of PARDS development in pediatric TBI.

Because neither the Trauma Quality Improvement Program nor the Brain Trauma Foundation guidelines address the utility of EtCO_2_ measurements in TBI, this study provides new evidence to fill this knowledge and practice gap. Our data show that, despite the relatively constant mean Paco_2_-EtCO_2_ difference and despite the moderate agreement correlation during the first 24 hours of PICU admission, there was even larger variability in the Paco_2_-EtCO_2_ difference in the first 8 hours of PICU admission compared with the subsequent 16 hours. Therefore, in addition to the overall observation that Paco_2_-EtCO_2_ agreement was low in the first 24 hours after TBI and in the presence of PARDS with large and variable alveolar dead space, EtCO_2_ may be even more unreliable as a surrogate for Paco_2_ during the first 8 hours after TBI. Therefore, serial Paco_2_ measurements during the first 24 hours of admission should still be considered the optimal modality to monitor CO_2_ levels, especially when ongoing resuscitation may be needed and optimizing cerebral perfusion is critical. Given the focus on prevention of prophylactic hypocarbia in severe pediatric TBI, these findings suggest a need for the Brain Trauma Foundation guidelines to address arterial measurements for Paco_2_.

### Strengths and Limitations

This study has several strengths, such as a relatively large sample size, a priori analytic framework, use of age-specific definitions of hypotension, examination of the association between Paco_2_-EtCO_2_ agreement and hypotension with temporal vicinity, inclusion of PARDS diagnosis as an explanatory variable based on laboratory and clinical information, and a comprehensive description of age subgroups. These methodical strengths enabled us to examine in greater detail the association of Paco_2_-EtCO_2_ agreement with hypotension and PARDS. In addition, compared with using severe chest trauma to investigate the association of respiratory disorders with Paco_2_-EtCO_2_ agreement, the use of the PARDS diagnosis provided more specific information regarding oxygenation dysfunction and resultant ventilation-perfusion mismatch. To our knowledge, this is the first analysis to effectively obtain timely data on blood pressure and PARDS diagnosis and to use them to examine Paco_2_-EtCO_2_ agreement.

We acknowledge the study limitations. Our findings might not be fully generalizable because the data are from a single level I trauma center and the excluded patients who did not have paired data points differed in several clinical characteristics. Although more than 40% of EtCO_2_ data points were recorded within 10 minutes of Paco_2_ measurement, some paired data points had a time gap of up to 30 minutes, which may have reduced the precision of our findings. We did not examine data beyond 24 hours. Despite using fixed-effects multivariable regression models and an a priori analytic framework, we cannot exclude residual confounding that could not be captured by measurable variables.

## Conclusions

In children hospitalized with TBI, Paco_2_-EtCO_2_ agreement is below 50%, especially in the presence of PARDS. Agreement is worst in the first 8 hours after PICU admission, and poor Paco_2_-EtCO_2_ agreement early in hospitalization may be associated with future development of PARDS. End-tidal CO_2_ data should not be a substitute for Paco_2_ data during the first 24 hours.

## Notes

Supplement. **eTable 1.** Characteristics of Study Cohort (n = 137) Compared With Those Who Were Excluded (n = 62) **eTable 2.** Fitted Regression Models and Covariates for the Factors Associated With Agreement Between Paco_2_ and EtCO_2_ **eTable 3.** Positive Predictive Values, Negative Predictive Values, Sensitivity and Specificity by Using Paco_2_-EtCO_2_ Difference in PICU Day 1 and Its Association With Subsequent Pediatric Acute Respiratory Distress Syndrome (PARDS) Development Between 1-7 days After PICU Admission **eFigure 1.** Example Sampling of PaCO_2,_ EtCO_2_ and Systolic Blood Pressure (SBP) **eFigure 2.** Histogram of the Number of Paco_2_-EtCO_2_ Pairs Per Patient **eFigure 3.** Passing and Bablok Regression Plot of PaCO_2_ and EtCO_2_ **eFigure 4.** Deming Regression Plot of PaCO_2_ and EtCO_2_ **eFigure 5.** Bland-Altman Plots of PaCO_2_ and EtCO_2_ Stratified by Age in Years **eFigure 6.** Side-by-Side Boxplots of Paco_2_-EtCO_2_ Differences Stratified by Patient
