# Supplementary material for: Agreement Between Arterial Carbon Dioxide Levels With End-Tidal Carbon Dioxide Levels and Associated Factors in Children Hospitalized With Traumatic Brain Injury
Source: JAMA Netw Open. 2019 Aug 16;2(8):e199448. doi: 10.1001/jamanetworkopen.2019.9448 (PMC6704750; doi:10.1001/jamanetworkopen.2019.9448)
Supplement: Supplement. — eTable 1. Characteristics of Study Cohort (n = 137) Compared With Those Who Were Excluded (n = 62) eTable 2. Fitted Regression Models and Covariates for the Factors Associated With Agreement Between Paco2 and EtCO2 eTable 3. Positive Predictive Values, Negative Predictive Values, Sensitivity and Specificity by Using Paco2-EtCO2 Difference in PICU Day 1 and Its Association With Subsequent Pediatric Acute Respiratory Distress Syndrome (PARDS) Development Between 1-7 days After PICU Admission eFigure 1. Example Sampling of PaCO2, EtCO2 and Systolic Blood Pressure (SBP) eFigure 2. Histogram of the Number of Paco2-EtCO2 Pairs Per Patient eFigure 3. Passing and Bablok Regression Plot of PaCO2 and EtCO2 eFigure 4. Deming Regression Plot of PaCO2 and EtCO2 eFigure 5. Bland-Altman Plots of PaCO2 and EtCO2 Stratified by Age in Years eFigure 6. Side-by-Side Boxplots of Paco2-EtCO2 Differences Stratified by Patient [file jamanetwopen-2-e199448-s001.pdf]

Supplementary Online Content

Yang J-T, Erickson SL, Killien EY, Mills B, Lele AV, Vavilala MS. Agreement between arterial carbon dioxide levels with end-tidal carbon dioxide levels and associated factors in children hospitalized with traumatic brain injury. *JAMA Netw Open.* 2019;2(8):e199448. doi:10.1001/jamanetworkopen.2019.9448

eTable 1. Characteristics of Study Cohort (n =137) Compared With Those Who Were Excluded (n=62)
eTable 2. Fitted Regression Models and Covariates for the Factors Associated With Agreement Between $PaCO_2$ and $EtCO_2$
eTable 3. Positive Predictive Values, Negative Predictive Values, Sensitivity and Specificity by Using $PaCO_2$-$EtCO_2$ Difference in PICU Day 1 and Its Association With Subsequent Pediatric Acute Respiratory Distress Syndrome (PARDS) Development Between 1-7 days After PICU Admission
eFigure 1. Example Sampling of $PaCO_2$, $EtCO_2$ and Systolic Blood Pressure (SBP)
eFigure 2. Histogram of the Number of $PaCO_2$-$EtCO_2$ Pairs Per Patient
eFigure 3. Passing and Bablok Regression Plot of $PaCO_2$ and $EtCO_2$
eFigure 4. Deming Regression Plot of $PaCO_2$ and $EtCO_2$
eFigure 5. Bland-Altman Plots of $PaCO_2$ and $EtCO_2$ Stratified by Age in Years
eFigure 6. Side-by-Side Boxplots of $PaCO_2$-$EtCO_2$ Differences Stratified by Patient

This supplementary material has been provided by the authors to give readers additional information about their work.

© 2019 Yang J-T et al. *JAMA Network Open.*

**eTable 1.** Characteristics of Study Cohort (n =137) Compared With Those Who Were Excluded (n=62)		
	**Study Cohort**	**Excluded**
n	137	62
**Age (yr), n (%)**		
**< 1**	8 (5.8)	11 (17.7)
**1 - 4**	32 (23.4)	13 (21.0)
**5 - 9**	21 (15.3)	6 (9.7)
**10 - 14**	15 (10.9)	9 (14.5)
**15 - 18**	61 (44.5)	23 (37.1)
**Male sex, n (%)**	103 (75.2)	38 (61.3)
**Mechanism, n (%)**		
**Motor vehicle crash**	46 (33.6)	20 (32.3)
**Fall**	30 (21.9)	19 (30.6)
**Struck by vehicle**	31 (22.6)	10 (16.1)
**Abusive TBI**	7 (5.1)	8 (12.9)
**Sport/recreation**	11 (8.0)	4 (6.5)
**Gunshot**	12 (8.8)	0 (0.0)
**Other**	0 (0.0)	1 (1.6)
**Head Abbreviated Injury Scale (AIS), median (IQR)**	5.00 [4.00, 5.00]	4.00 [4.00, 5.00]
**Chest Abbreviated Injury Scale (AIS), median (IQR)**	3.00 [2.00, 3.00]	3.00 [2.00, 3.00]
**Injury Severity Score (ISS), median (IQR)**	30.00 [25.00, 38.00]	26.00 [17.00, 33.00]
**Polytrauma, n (%)**	67 (48.9)	19 (30.6)
**ED admit Glasgow Coma Scale score, median (IQR)**	3.00 [3.00, 6.00]	3.00 [3.00, 6.00]
**ED Glasgow Coma Scale (motor), median (IQR)**	1.00 [1.00, 1.00]	1.00 [1.00, 1.00]
**PICU admit Glasgow Coma Scale score, median (IQR)**	6.50 [3.00, 8.00]	10.00 [8.00, 13.00]
**Discharge Glasgow Coma Scale score (motor), median (IQR)**	6.00 [6.00, 6.00]	6.00 [6.00, 6.00]
**Any surgery, n (%)**	74 (54.0)	48 (77.4)
**Craniotomy, n (%)**	46 (33.6)	10 (16.1)
**Intracranial pressure monitoring, n (%)**	62 (45.3)	10 (16.1)
**Experienced brain herniation, n (%)**	63 (46.0)	15 (24.2)
**Discharge survival, n(%)**	103 (75.2)	57 (91.9)
TBI = Traumatic Brain Injury, GCS = Glasgow Coma Score, ED = Emergency Department		

© 2019 Yang J-T et al. *JAMA Network Open.*

eTable 2. Fitted Regression Models and Covariates for the Factors Associated With Agreement Between $PaCO_2$ and $EtCO_2$

Covariates for OR of PARDS within 24 hours of PICU admission, head injury severity, chest injury severity, maximum non-head non-chest injury severity

	**Estimate**	**Lower Limit**	**Upper Limit**
Intercept	0.335426	0.060519	1.859078
PARDS within 24 hours of PICU admission	0.199728	0.078236	0.509883
$PaCO_2$-$EtCO_2$ pair during hypotension	1.118286	0.571751	2.187255
severe chest injury	1.434578	0.809199	2.543272
1 – 4 years of age	1.466849	0.447627	4.806787
5 – 9 years of age	1.097218	0.30676	3.924523
10 – 14 years of age	1.789781	0.504194	6.353345
15 – 18 years of age	1.593966	0.513088	4.951838
severe head injury	1.494089	0.415851	5.368037
severe non head, non chest injury	1.14226	0.660425	1.975633

Covariates for OR of PARDS 1 – 7 days after PICU admission

	**Estimate**	**Lower Limit**	**Upper Limit**
Intercept	0.238089	0.02787	2.033937
PARDS 1-7 days after PICU admission	1.224181	0.611668	2.450055
$PaCO_2$-$EtCO_2$ pair during hypotension	1.228887	0.578263	2.611549
severe chest injury	1.579863	0.831677	3.001127
1 – 4 years of age	1.347879	0.41099	4.420489
5 – 9 years of age	1.172141	0.321375	4.275111
10 – 14 years of age	2.093238	0.584019	7.502574
15 – 18 years of age	1.607609	0.51561	5.012333
severe head injury	1.967799	0.3209	12.06681
severe non head, non chest injury	1.068462	0.606541	1.882167

Covariates for OR of Hypotension status of $PACO_2$-$EtCO_2$ pair with fixed effect

	**Estimate**	**Lower Limit**	**Upper Limit**
$PaCO_2$-$EtCO_2$ pair during hypotension	0.675	0.3024	1.507

© 2019 Yang J-T et al. *JAMA Network Open.*

eTable 3. Positive Predictive Values, Negative Predictive Values, Sensitivity and Specificity by Using $PaCO_2$-$EtCO_2$ Difference in PICU Day 1 and Its Association With Subsequent Pediatric Acute Respiratory Distress Syndrome (PARDS) Development Between 1-7 days After PICU Admission

Paired data points for this table were from patients who had not developed PARDS within the first 24 hours.

**$PaCO_2$ – $EtCO_2$ difference cutoff (mmHg)**	**TP / FP / FN / TN**	**PPV**	**NPV**	**Sensitivity**	**Specificity**
≥1	76 / 129 / 16 / 157	0.37	0.91	0.83	0.55
≥2	71 / 96 / 21 / 190	0.43	0.90	0.77	0.66
≥3	66 / 80 / 26 / 206	0.45	0.89	0.72	0.72
≥4	55 / 53 / 37 / 233	0.51	0.86	0.60	0.81
≥5	40 / 34 / 52 / 252	0.54	0.83	0.43	0.88
≥6	34 / 26 / 58 / 260	0.57	0.82	0.37	0.91
≥7	29 / 18 / 63 / 268	0.62	0.81	0.32	0.94
≥8	19 / 14 / 73 / 272	0.58	0.79	0.21	0.95
≥9	15 / 11 / 77 / 275	0.58	0.78	0.16	0.95
≥10	7 / 10 / 85 / 276	0.41	0.76	0.08	0.97

TP = true positive, FP = false positive, FN = false negative, TN = true negative,

PPV = positive predictive value, NPV = negative predictive value

© 2019 Yang J-T et al. *JAMA Network Open.*

eFigure 1. Example Sampling of $PaCO_2$, $EtCO_2$ and Systolic Blood Pressure (SBP)

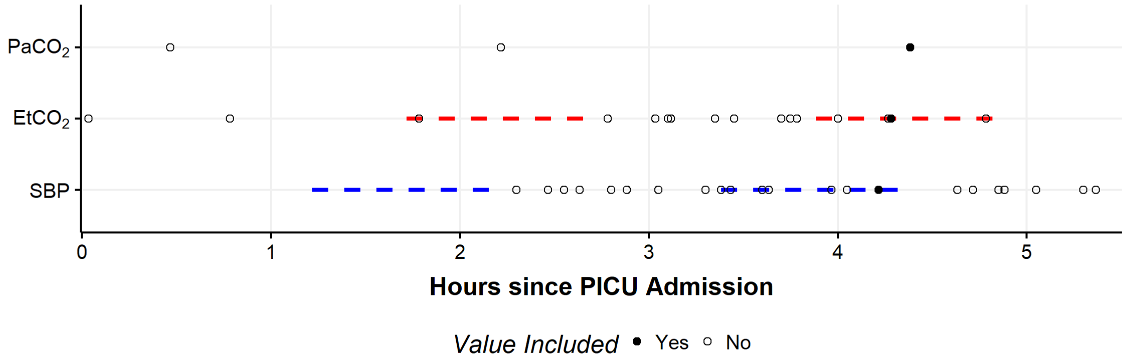

Plot shows all $PaCO_2$ (n = 3), $EtCO_2$ (n = 31) and systolic blood pressure values (n = 30) for one patient during the first 5 hours of PICU admission. $PaCO_2$ was aligned to the most proximally recorded EtCO2 within 30 minutes before or after the time of blood draw (example ranges highlighted in red). $PaCO_2$ was aligned to the most proximally recorded SBP value 0 to 60 minutes prior to the time of blood draw (example ranges highlighted in blue). $PaCO_2$ values that were not able to be paired with both an EtCO2 value and an SBP value were not included in final analysis. In this example, two $PaCO_2$ values were discarded (unfilled circles) as they were unable to be paired with a SBP value. In our study, of the 62 patients who were excluded, 25 patients lacked a recorded $EtCO_2$ value, 17 patients lacked an $EtCO_2$ recorded within 30 minutes of a $PaCO_2$ value, and 20 patients lacked SBP data within 60 minutes prior to a $PaCO_2$ value.

© 2019 Yang J-T et al. *JAMA Network Open.*

eFigure 2. Histogram of the Number of $PaCO_2$-$EtCO_2$ Pairs Per Patient

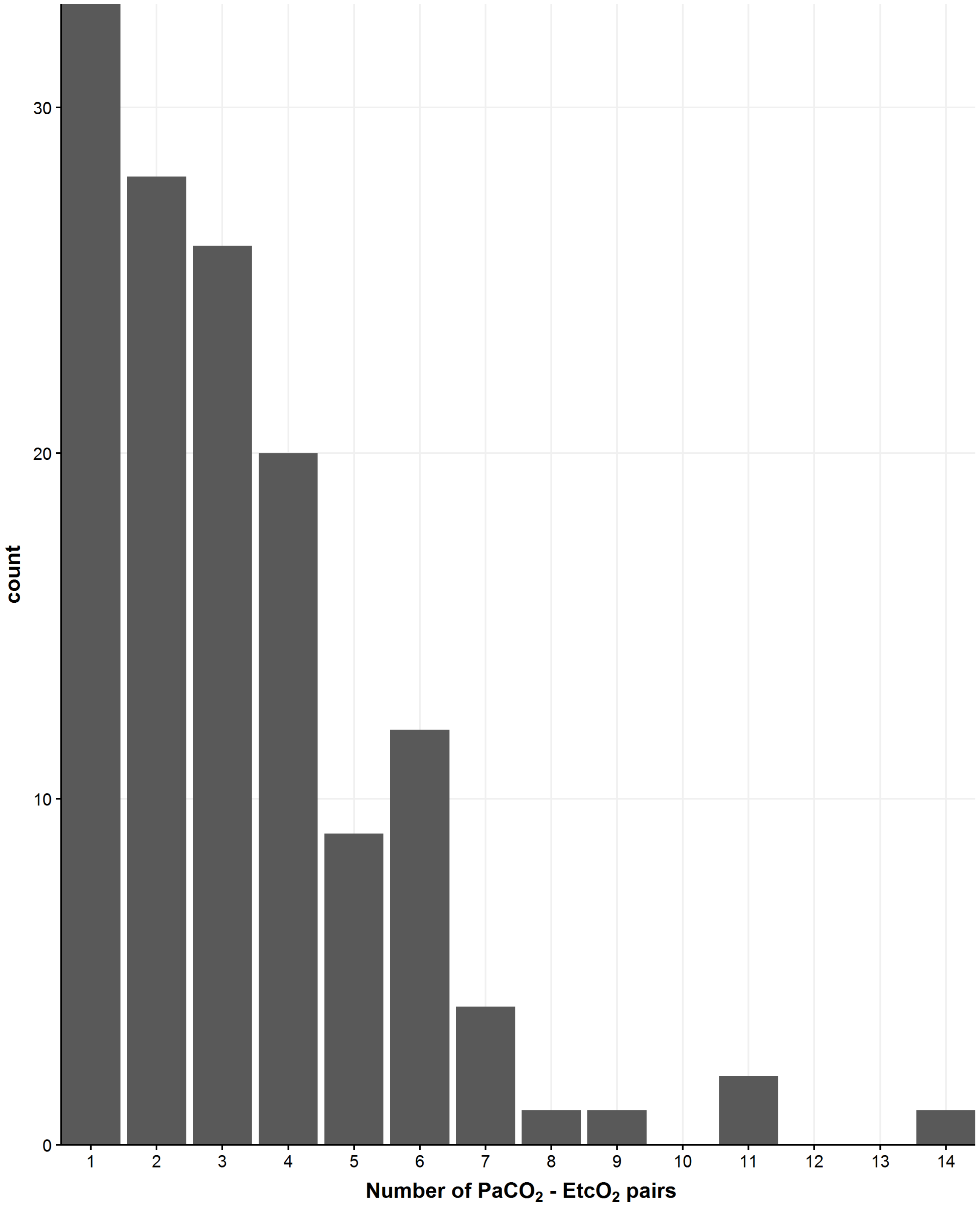

Data represents 445 $PaCO_2$-$EtCO_2$ pairs for 137 patients.

© 2019 Yang J-T et al. *JAMA Network Open.*

eFigure 3. Passing and Bablok Regression Plot of $PaCO_2$ and $EtCO_2$

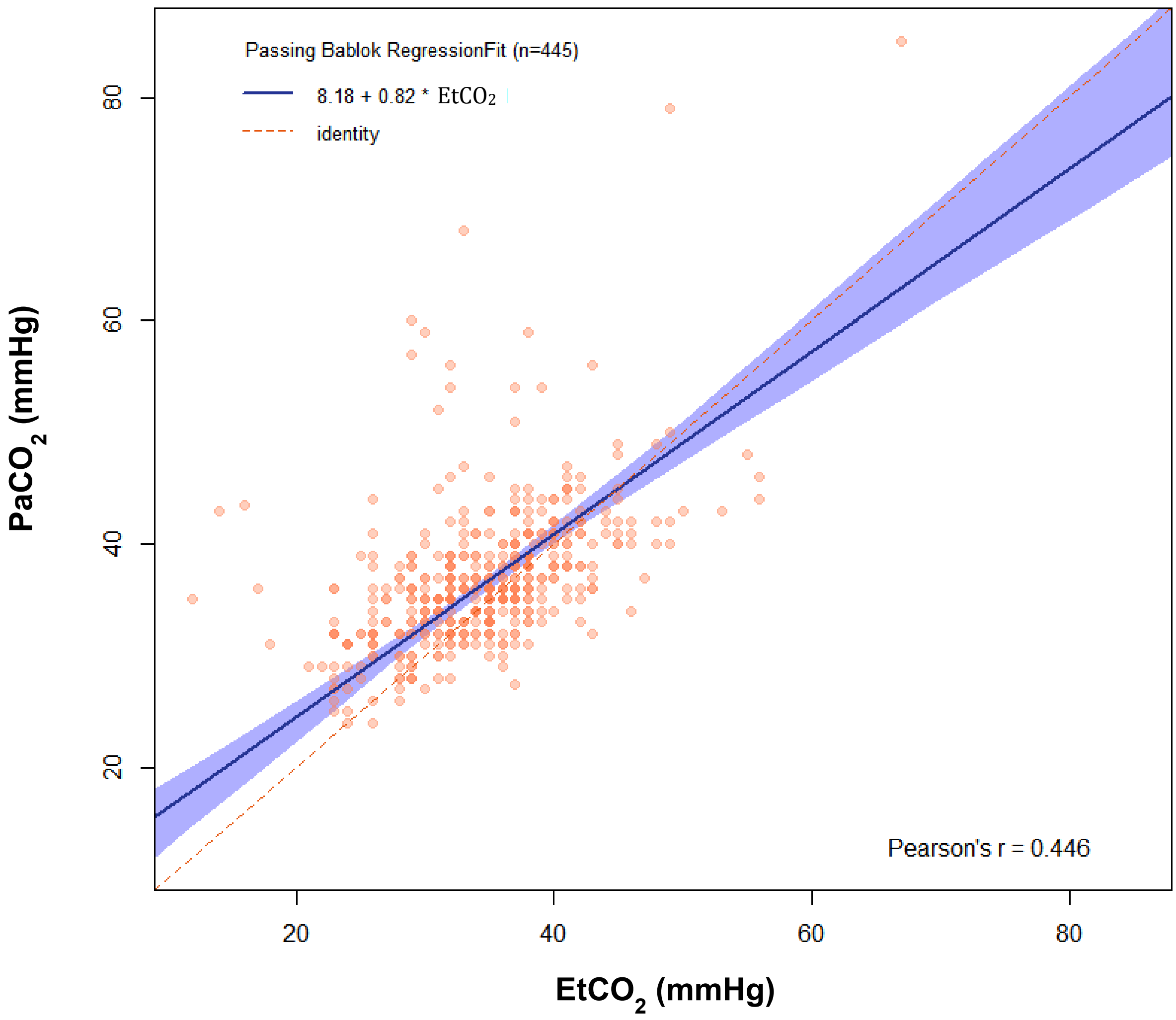

Red dots represent the 445 $PaCO_2$ - $EtCO_2$ pairs for 137 unique patients. Blue line represents the fitted Passing and Bablok regression line. Red dotted line indicates a theoretical fitted Passing and Bablok line with no absolute constant difference between measures (intercept of zero), and no proportional difference between measures (slope of one). $EtCO_2$ demonstrated the highest degree of interchangeability with $PaCO_2$ at 45.44mmHg; with a proportional difference of 0.81mmHg (95% CI 0.72 – 1.00) of $PaCO_2$ per 1mmHg of $EtCO_2$.

© 2019 Yang J-T et al. *JAMA Network Open.*

eFigure 4. Deming Regression Plot of $PaCO_2$ and $EtCO_2$

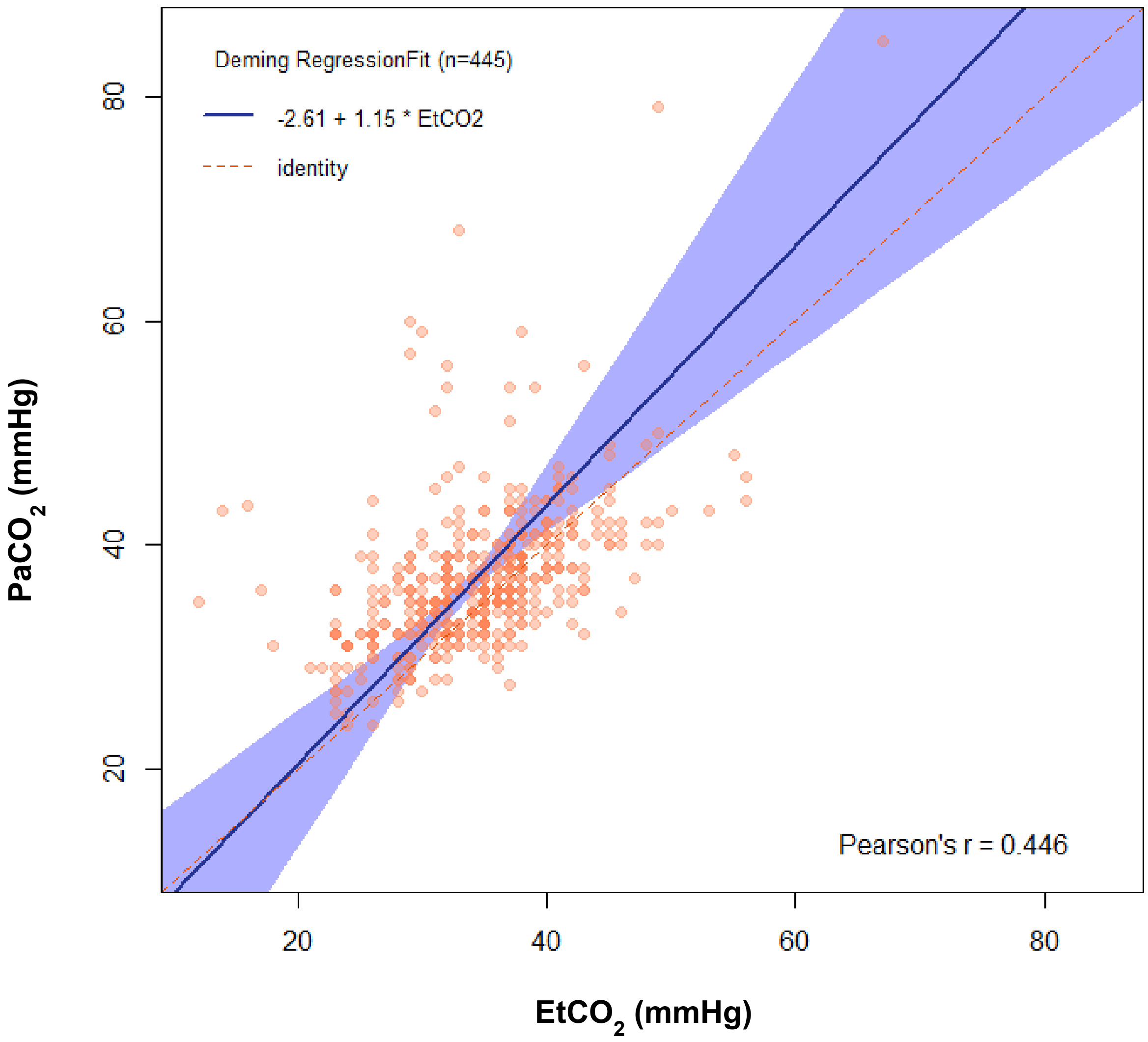

Red dots represent the 445 $PaCO_2$ - $EtCO_2$ pairs for 137 unique patients. Blue line represents the Deming regression line. Red dotted line indicates a theoretical fitted Demming line with no absolute constant difference between measures (intercept of zero), and no proportional difference between measures (slope of one).

© 2019 Yang J-T et al. *JAMA Network Open.*

eFigure 5. Bland-Altman Plots of $PaCO_2$ and $EtCO_2$ Stratified by Age in Years

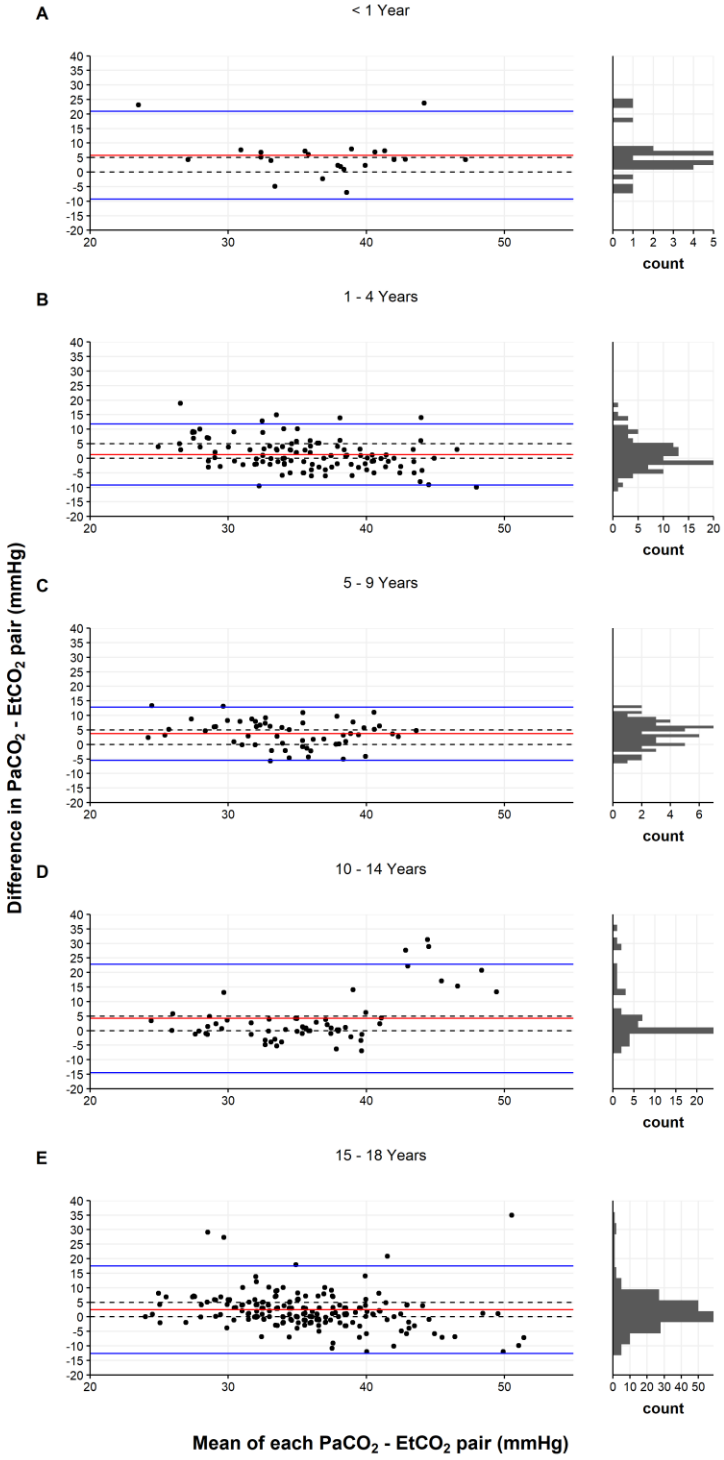

© 2019 Yang J-T et al. *JAMA Network Open.*

Bias (average PaCO2-EtCO2 difference) is represented by the red line. The limits of agreement are represented by the blue lines and adjusted for repeated measures. *A priori* limits of acceptable agreement are represented by the dashed lines. Corresponding marginal histograms describe distribution of values between pairs. 5A contains 23 paired $PaCO_2$-$EtCO_2$ pairs for 8 patients, 9 (39%) of these pairs had $EtCO_2$ values within 5mmHg of paired $PaCO_2$. 5B contains 113 paired $PaCO_2$-$EtCO_2$ pairs for 32 patients, 44 (39%) of these pairs had $EtCO_2$ values within 5mmHg of paired $PaCO_2$. 5C contains 56 paired $PaCO_2$-$EtCO_2$ pairs for 21 patients, 19 (34%) of these pairs had $EtCO_2$ values within 5mmHg of paired $PaCO_2$. 5D contains 60 paired $PaCO_2$-$EtCO_2$ pairs for 15 patients, 29 (48%) of these pairs had $EtCO_2$ values within 5mmHg of paired $PaCO_2$. 5E contains 193 paired $PaCO_2$-$EtCO_2$ pairs for 61 patients, 86 (45%) of these pairs had $EtCO_2$ values within 5mmHg of paired $PaCO_2$.

© 2019 Yang J-T et al. *JAMA Network Open.*

eFigure 6. Side-by-Side Boxplots of $PaCO_2$-$EtCO_2$ Differences Stratified by Patient

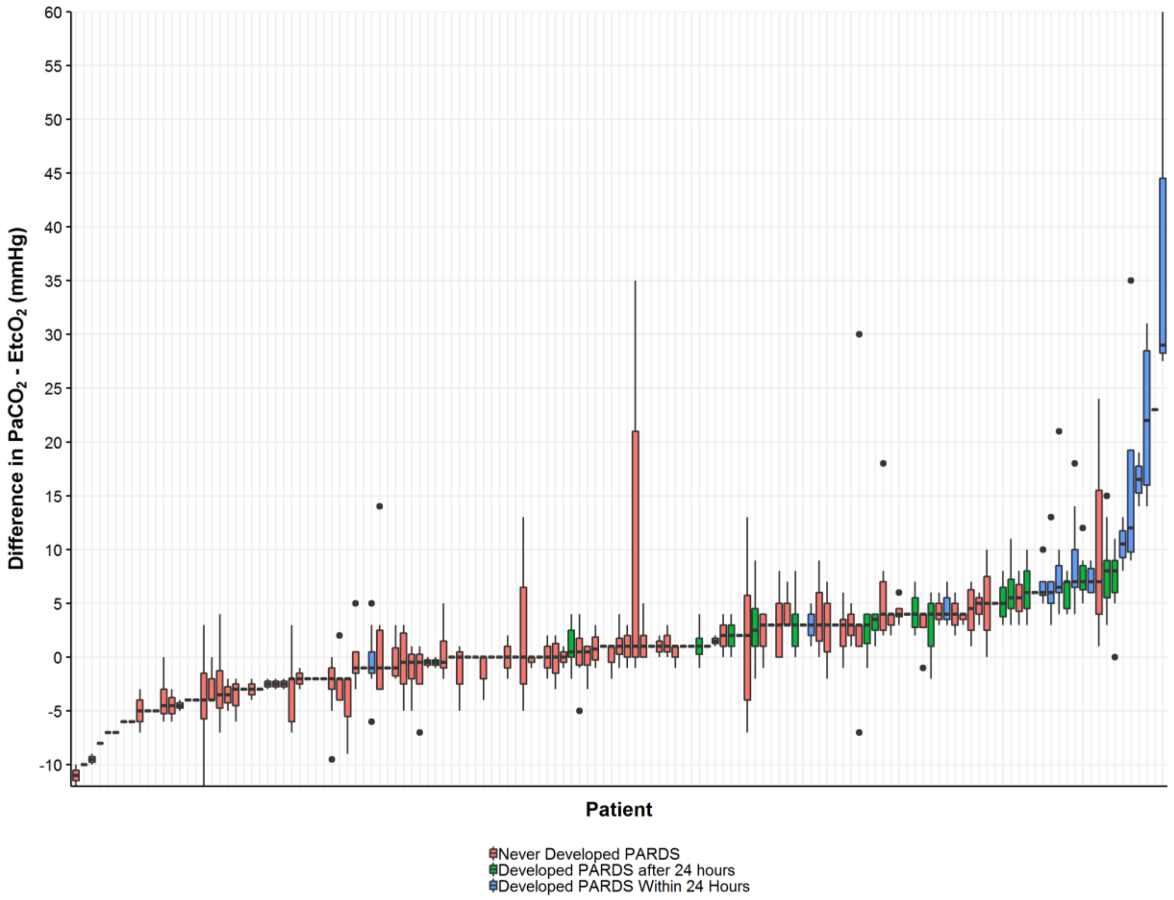

Plotted data represents all 445 $PaCO_2$-$EtCO_2$ pairs obtained within 24 hours of Pediatric Intensive Care Unit (PICU) admission for 137 PEGASUS program patients. Center horizontal line indicates median $PaCO_2$-$EtCO_2$ difference for each patient value. Top and bottom of the boxes represent 75th percentile and 25th percentile of $PaCO_2$-$EtCO_2$ difference for each patient, whiskers represent the median plus and minus 1.5 times the interquartile range. Circles represent all values beyond the median + 1.5 times the interquartile range. Colors represent the timing of Pediatric Acute Respiratory Distress Syndrome (PARDS) development, with those who never developed PARDS in red (n = 101, pairs = 286), those who developed PARDS after 24 hours from PICU admission (n = 22, pairs = 92), and those who developed PARDS within 24 hours from PICU admission (n = 14, pairs = 67).

© 2019 Yang J-T et al. *JAMA Network Open.*
